# Probiotics Reduce Inflammation in Antiretroviral Treated, HIV-Infected Individuals: Results of the “Probio-HIV” Clinical Trial

**DOI:** 10.1371/journal.pone.0137200

**Published:** 2015-09-16

**Authors:** Gabriella d’Ettorre, Giancarlo Ceccarelli, Noemi Giustini, Sara Serafino, Nina Calantone, Gabriella De Girolamo, Luigi Bianchi, Valeria Bellelli, Tommaso Ascoli-Bartoli, Sonia Marcellini, Ombretta Turriziani, Jason M. Brenchley, Vincenzo Vullo

**Affiliations:** 1 Department of Public Health and Infectious Diseases, University of Rome “Sapienza”, Rome, Italy; 2 Department of Virology, University of Rome “Sapienza”, Rome, Italy; 3 Program in Barrier Immunity and Repair, Lab of Molecular Microbiology, NIAID, NIH, Bethesda, Maryland, United States of America; Rush University, UNITED STATES

## Abstract

**Background:**

HIV infection results in damage to the gastrointestinal (GI) tract, microbial translocation and immune activation. These are not completely normalized with combined antiretroviral therapy (cART). Moreover, increate morbidity and mortality of cART-treated HIV-infected individuals is associated with inflammation.

**Methods:**

In order to enhance GI tract immunity, we recruited and treated 20 HIV-infected humans with cART supplemented with probiotics and followed inflammation and immunological parameters (clinical trial number NCT02164344). 11 HIV seronegative subjects were included as control group. The enumeration of CD4+, CD8+, CD38+ and HLA-DR+ lymphocytes were evaluated on peripheral blood; HIV-RNA levels, sCD14, d-dimer, C-reactive protein (CRP) high sensitivity C-reactive protein (hsCRP), IL-6 and Lipopolysaccharide Binding Protein (LBP) were assayed on plasma.

**Results:**

We observe that cART does not normalize the levels of immune activation in HIV positive patients anyway inflammation and markers of microbial translocation were significantly reduced with probiotic supplementation. Patients show a clear and statistically significant reduction in the levels of immune activation on CD4 T-lymphocytes, for both markers CD38 and HLA-DR and their simultaneous expression, LBP and hsCRP plasma levels after probiotic diet supplementation settling to values comparable to controls.

**Conclusions:**

Supplementing cART with probiotics in HIV-infected individuals may improve GI tract immunity and there by mitigate inflammatory sequelae, ultimately improving prognosis.

**Trial Registration:**

ClinicalTrials.gov NCT02164344

## Introduction

The life expectancy of HIV infected patients has dramatically changed after the introduction of combined antiretroviral therapy (cART). However cART-treated patients still have increased mortality and morbidity compared to age-matched seronegative individuals which involves cardiovascular disease, neurocognitive disorders, liver and kidney disease and some cancers [1]. This increased mortality and morbidity has been associated to immune activation that persists also in patients under cART even with undetectable levels of HIV-RNA in blood [2–4]. Indeed HIV-infected patients show higher levels of activated T cells, inflammatory monocytes and proinflammatory cytokines than seronegative individuals, irrespective of cART [5–6]. Several putative causes of this residual inflammation have been proposed and include ongoing HIV replication at low levels, the presence of coinfections, and microbial translocation. For these reasons the consequent residual inflammation in cART treated individuals will require novel therapeutic interventions aimed at alleviated each putative cause [7–19]. Here we aim to reduce immune activation related to microbial translocation with supplementation of cART with a probiotic mixture. Indeed, the early stage of HIV infection is associated with dysbiosis of the GI tract microbiome with reduced levels of bifidobacteria and lactobacillus species with increased levels of potentially pathogenic proteobacteria species. We conducted a longitudinal study in HIV infected patients in cART and with persistent undetectable plasma levels of HIV-RNA to investigate the potential benefits of 48 weeks of probiotics supplementation on immune function and on immune activation status.

## Material and Methods

### Study design, recruitment and study eligibility criteria

**Fig 1:**
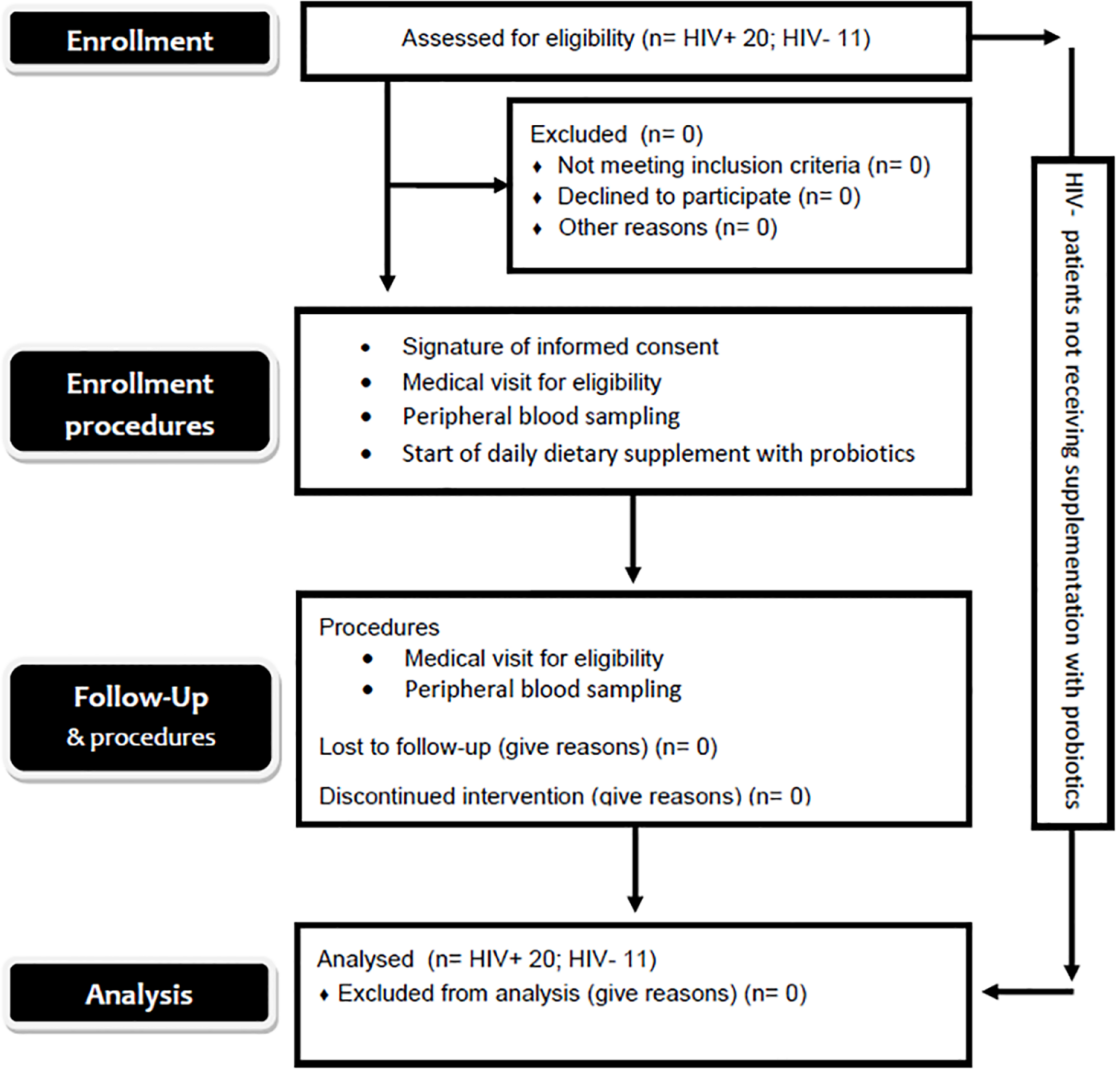
The CONSORT flow diagram of the clinical trial. Experimental design of the study.

The protocol for this trial and supporting TREND checklist are available as supporting information; see S1 TREND Checklist, S1 Protocol (Protocol in Italian), and S2 Protocol (Protocol in English). This is a longitudinal pilot study (Fig 1). This study, named “Probio-HIV”, is registered on the ClinicalTrials.gov registry with identifier number NCT02164344. Study participants included a total of 20 HIV-infected patients and 11 healthy donors enrolled from January 2013 to September 2013 from the Department of Public Health and Infectious Diseases of the University of Rome“Sapienza”.

The inclusion criteria allowed to enroll HIV-positive patients who have signed the informed consent and aged between 18 and 80 years. Moreover, in order to avoid the influence of previous cART exposure, active viral replication and switches of treatment, the eligibility criteria allowed to enrol patient under cART with persistent undetectable levels of HIV-RNA (detection limit of viremia in routinely clinical monitoring of the patients: 37 copy/mL measured with Versant kPCR) and without history of drugs failure. Other inclusion criteria included: i) no change in antiretroviral therapy since the beginning of the cARV treatment, ii) no history of AIDS-defining conditions from the diagnosis of HIV to the enrolment iii) no current diagnosis of acute infection.

At baseline (T0) the evaluation of immune activation of all subjects was performed. HIV-1 infected patients were then asked to take daily dietary supplement with probiotics (1 g packet containing *Streptococcus salivarius* ssp. *Termophilus* [at least 204 billion Colony Forming Unit—CFU], Bifidobacteria–represented by *B*. *breve*, *B*. *infantis* and *B*. *longum* [at least 93 billion CFU], *Lactobacillus acidophilus* [at least 2 billion CFU], *Lactobacillus plantarum* [at least 220 million CFU], *Lactobacillus casei* [220 million CFU], *Lactobacillus delbrueckii* ssp. *Bulgaricus* [at least 300 million CFU] and *Streptococcus faecium* [at least 30 million CFU]) for 48 weeks, twice a day. A second evaluation of their immune activation was performed 48 weeks after the baseline analysis (T1). The healthy subjects did not consume probiotics, and were used as controls of treatment group to compare immune activation levels.

A self-administered questionnaire was fulfilled by participants at the end of probiotics’ intake to investigate their effects on bowel activity.

### Ethics Statement

The study was approved by the institutional review board (Department of Public Health and Infectious Diseases, University of Rome ‘‘Sapienza” and Ethics Committee Azienda Policlinico Umberto I of Rome). All study participants gave informed written informed consent and the research was conducted in accordance with the Helsinki Declaration of 1975 and revised in 2000. The approval of the independent Ethics Committee is the only form of registration required by Italian law; for this reason, the registration in an international registry was made after patient recruitment. Moreover the authors confirm that all ongoing and related trials for this intervention are registered.

#### CD4+, CD8+, CD38+ and HLA-DR+ lymphocyte counts

Peripheral blood samples were collected in tubes containing ethylenediaminetetraacetic acid and Lyse/Wash Procedure was applied; lymphocytes T cells enumeration and activation were evaluated by the following anti-human monoclonal antibodies: CD3 VioBlue, CD 4 APC-Vio770, CD8 FITC, CD38 APC and HLA-DRPE (MiltenyiBiotec). 5-color flow cytometric analysis was performed with the MiltenyiBiotec flow citometer-MACSQuantAnalyzer (8 fluoresces, 3 lasers). Gating analysis was developed with MACSQuantify software 2.5 (MiltenyiBiotec) with the same gating strategy applied to all samples. We tested specificity for the expression of CD38 and HLA-DR markers, by using Fluorescence Minus One method (FMO): each cell population was stained with all the fluorescence antibodies, except one, at a time, in order to verify whether in the absence of one antibody, the labelled cells were negative for the removed one; this method allows the subset to be gated and the background level for the subset to be determined.

#### Plasma markers of microbial translocation and immune activation

EDTA plasma was used to perform Enzyme linked Immunosorbent Assays. Commercially available ELISAs were used to quantify plasma levels of sCD14 (R&D Systems, Minneapolis, MN, detection limit 125 pg/mL), D-dimer (Wuhan Hubei Province, China, detection limit 3.9 ng/mL), C-reactive protein (R&D Systems, Minneapolis, MN, detection limit 23 pg/mL), hsCRP (BioVendor R&D, Torino, detection limit 0.02 μg/mL), IL-6 (R&D Systems, Minneapolis, MN, detection limit 1 pg/mL) and LBP (Enzo Life Sciences, Florence, detection limit 25 ng/mL). All were performed according to the manufacturer’s protocols and absorbances were read at 450 nm with an automated Microplate Reader (BIO-RAD).

### HIV-RNA levels

HIV-RNA levels were measured in plasma prepared from blood obtained in EDTA-containing tubes and stored at -70°C. A quantitative reverse polymerase chain reaction was measured with Versant kPCR (Siemens Healthcare Diagnostics). The limit of detection was 1 copy/mL.

#### Questionnaires

A self-administered questionnaire was fulfilled by participants at the end of the study to investigate probiotics’ actual intake and their effects on bowel activity.

The questionnaire included personal data such as age, level of education and work; it was administered to assess the duration and the intake period of probiotics, the regularity with which such an assumption had been made and benefits/inconvenience perceived; since the purchase was charged to the patient, we asked if the cost has been excessive. We also inquired if in the past they had already made use of lactic acid bacteria.

#### Statistical analysis

Statistical analysis were performed with SigmaStat software (version 2.03). Prior to the statistical analyses, normal distribution and homogeneity of the variances were tested. Values are given as mean and standard deviation (SD). For comparison of the immune activation between the two different times of analysis, we used Paired t-test or Wilcoxon signed-rank test when the data showed a non-Gaussian distribution; to compare the data with the control group a simple t-test (Mann Whitney U-test for skewed distributed variables) was performed. and correlations were determined by nonparametric Spearman's rank test. P values <0.05 were considered statistically significant.

All graphs were generated by using GraphPad Prism (version 5.00) and SigmaPlot software (version 8.0).

## Results

### Patient population

20 HIV-1 infected individuals, 3 women and 17 men, were recruited and underwent a blood collection. The median age was 54 years (range 27–72). All subjects were on cART from at least 3 years (median 12,5 years; range 3–20) and had a median of CD4+ T cell count of 542 cells/μl (range 285–1402) and HIV RNA <50 copies/mL at the moment of enrolment.11 HIV seronegative subjects were included as control group, age ranging from 28 to 73 years with a median age of 43 (7 women and 4 men).

Table 1 shows the basic demographic, immunological and virological characteristics of the 20 HIV-infected patients who were included in the study. Tables 2 and 3 show respectively the changes in value of cellular activation levels and of plasma cytokines concentrations from T0 to T1 in HIV population. Moreover Table 3 compares also the T1 values registered in HIV+ patients with those observed in healthy controls. There were no adverse events in any of subjects enrolled; furthermore none patients required modification to their treatment regimen and none reported clinical progression.

**Table 1:** Demographic, immunological and virological characteristics of the 20 HIV-infected patients and 11 controls included in the study.

Pts&Ctrs	Age (Yrs)	Sex	TherapyCurrent	CD4+(Cell/μL) Nadir	CD4+(Cell/μL) T0	CD4+% T0	CD4+(Cell/μL) T1	CD4+% T1	HIV-RNA (copies/mL) T0	HIV-RNA (copies/mL) T1
**P1**	49	M	treated	300	550	19,07	727	25,67	36	37
**P2**	47	M	treated	63	426	12,88	595	20,46	22	1
**P3**	68	M	treated	300	579	22,52	691	24,57	1	1
**P4**	59	F	treated	50	767	39,12	714	36,91	1	1
**P5**	67	M	treated	16	329	7,47	429	14,37	1	1
**P6**	55	M	treated	70	608	21,38	455	17,51	1	1
**P7**	27	M	treated	50	394	14,34	389	17,85	1	1
**P8**	57	F	treated	50	527	23,25	348	19	50	29
**P9**	44	M	treated	300	1402	33,88	1244	36,1	1	1
**P10**	49	M	treated	20	467	23,01	446	25,91	1	9
**P11**	46	M	treated	29	629	33,78	672	34,21	8	30
**P12**	64	M	treated	300	897	36,86	862	34,81	1	1
**P13**	57	M	treated	350	597	24,07	686	26,03	13	1
**P14**	72	M	treated	50	285	16,4	180	18,01	9	1
**P15**	51	F	treated	180	833	21,84	897	27,38	1	1
**P16**	53	M	treated	69	402	21,75	454	28,46	1	22
**P17**	72	M	treated	280	533	43,16	581	39,15	1	1
**P18**	44	M	treated	150	640	37,89	503	36,82	1	1
**P19**	40	M	treated	400	513	43,63	825	44,77	16	1
**P20**	57	M	treated	200	530	32,69	638	33,92	1	1
**C1**	32	M	untreated	-	-	57,87	-	-	0	0
**C2**	29	F	untreated	-	-	48,21	-	-	0	0
**C3**	51	F	untreated	-	-	61,59	-	-	0	0
**C4**	43	M	untreated	-	-	38,49	-	-	0	0
**C5**	73	F	untreated	-	-	40,96	-	-	0	0
**C6**	28	F	untreated	-	-	50,24	-	-	0	0
**C7**	44	F	untreated	-	-	55,45	-	-	0	0
**C8**	50	F	untreated	-	-	50,74	-	-	0	0
**C9**	36	F	untreated	-	-	65,74	-	-	0	0
**C10**	49	M	untreated	-	-	72,17	-	-	0	0
**C11**	28	M	untreated	-	-	68,54	-	-	0	0

**Table 2:** Patients and Controls’ CD4 and CD8 immune activation markers’ mean values, standard deviations and T-test p-values.

	MEAN T0	STD. DEV. T0	MEAN T1	STD. DEV. T1	PAIRED T-TEST p-values	MEAN CONTROLS	STD. DEV. CONTROLS	T-TEST p-values controls/HIV+ T0	T-TEST p-values controls/HIV+ T1
**CD4+CD38+HLA-DR-%**	17,24	11,84	11,23	8,49	**0,004**	20,93	12,31	0,42	**0,015**
**CD4+CD38-HLA-DR+%**	11,18	8,58	8,2	6,65	**0,001**	4,05	2,45	**<0,001**	**0,008**
**CD4+CD38+HLA-DR+%**	2,57	1,42	1,24	0,68	**<0,001**	1,28	1,26	**0,002**	0,47
**CD8+CD38+HLA-DR-%**	4,98	3,63	3,29	4,54	0,237	3,31	1,84	0,166	0,143
**CD8+CD38-HLA-DR+%**	14,17	11,16	13,23	9,41	0,608	7,26	4,57	0,06	**0,026**
**CD8+CD38+HLA-DR+%**	5,23	4,45	2,78	2,28	**0,015**	2,84	5,17	**0,002**	0,208

**Table 3:** Plasma markers’ mean values, standard deviation and t-test p-values at both analysis time in patients and their comparison with controls.

	MEAN T0	STD. DEV. T0	MEAN T1	STD. DEV. T1	PAIRED T-TEST p-values	MEAN CONTROLS	STD. DEV. CONTROLS	T-TEST p-values controls/HIV+ T0	T-TEST p-values controls/HIV+ T1
**sCD14 ng/mL**	1941,59	492,29	1925,74	507,74	0,812	1296,92	309,29	**<0,001**	**<0,001**
**d-dimer ng/mL**	93,8	148,98	79,44	125,91	0,182	7,58	8,54	**0,008**	**0,024**
**CRP ng/mL**	2677,51	2468,92	1723,74	1246,68	0,095	1685,68	1630,40	0,243	0,942
**hsCRP mg/L**	3,36	2,95	1,62	1,42	**0,006**	1,08	1,33	**0,022**	**0,186**
**IL-6 pg/mL**	3,89	5,14	2,22	3,80	**0,027**	1,02	0,63	0,291	0,885
**LBP μg/mL**	26,94	6,16	25,4	4,48	0,266	22,52	3,07	**0,035**	**0,068**

#### cART does not normalize the levels of immune activation

As expected, the percentage levels of CD4+ T cells in the sample are significantly lower compared to healthy controls, respectively 26.45±10.46 (media±SD) and 55.45±10.95 (p<0.001). The levels of immune activation instead were higher in HIV+ patients than in healthy controls (CD4+/CD38-/DR+ in HIV+ patients and healthy controls, were respectively 11.18±8.58 vs 4.05±2.46, p<0.001).

The double activated subsets, both CD4+/CD38+/HLA-DR+ and CD8+/CD38+/HLA-DR+ show in HIV+ levels strongly higher, respectively 2.57±1.42 and 5.23±4.45, compared with those in healthy donors (1.28±1.26 and 2.84±5.17, p = 0.002).

#### Impact of probiotics on CD4 T-cells

A weak increase in CD4 percentages and absolute numbers among HIV-1 infected patients between T0 and T1 was observed (respectively 26.45±10.46 and 28.1±8.62, p = 0.065) with median recovery of 65 cell/μL.

### Changes in markers of immune activation during the 48 weeks of probiotic diet supplementation

In this analysis, the levels of immune activation were compared between cART-treated HIV-infected individuals before probiotics (T0), and after probiotic intake (T1). Comparing T1 to T0, the percentage of CD4+CD38+HLA-DR+ T cells was significantly lower (respectively 1.24±0.68 and 2.57±1.42, p<0.001). The same trend was observed in CD8+CD38+HLA-DR+ T cells, patients showed a percentage of 5.23±4.45 before and a percentage of 2.78±2.28 after lactic acid bacteria intake (p = 0.015).

**Fig 2:**
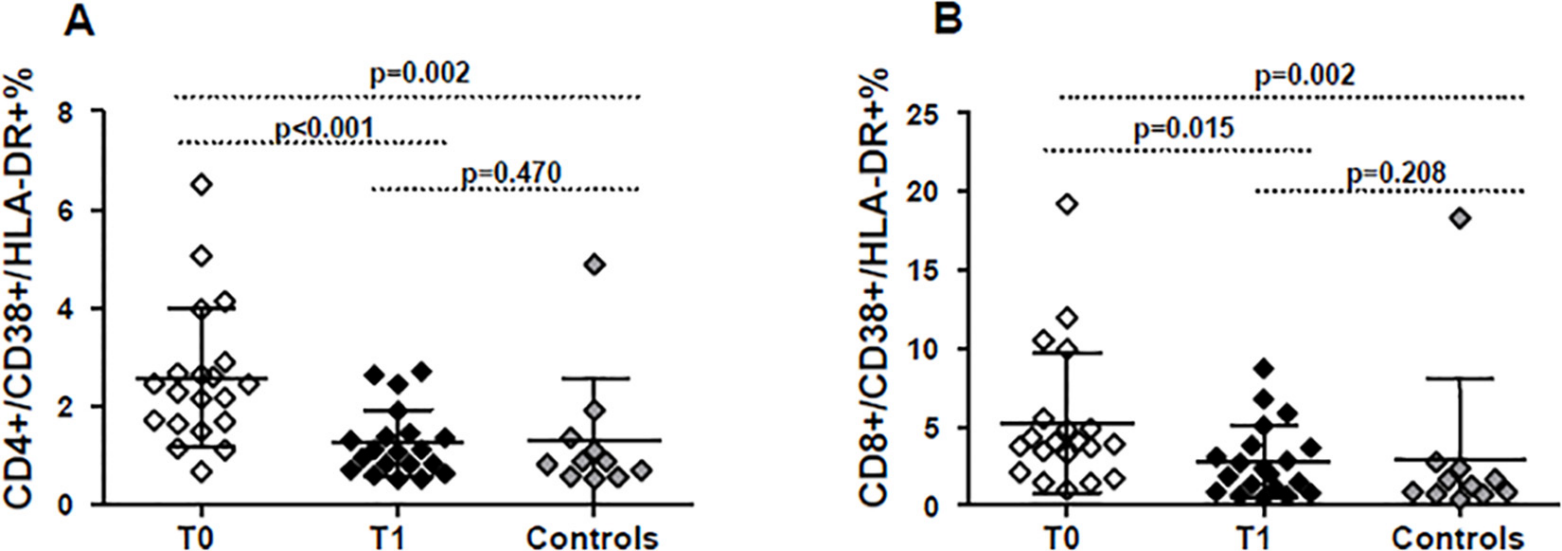
Immune activation on T-lymphocytes. Longitudinal assessment of activation markers(CD38+HLA-DR+) on CD4+ cells (A) and on CD8+cells (B)before (T0) and after (T1) probiotic diet supplementation. Control group was added for comparison. Horizontal bars in the scatter plot represent mean value with SD. P values <0.05 were considered statistically significant.

Moreover, despite a significantly higher percentage of CD4+CD38+HLA-DR+ and CD8+CD38+HLA-DR+ T cells observed at T0 in HIV-1 infected subjects compared to HIV seronegative donors (p = 0.002), the expression of activation markers on CD4 and CD8 surface at T1 resulted comparable to healthy donors (Fig 2).

No correlation was found between patients’ CD4 nadir and the CD4 recovery (ΔCD4), both T0 and T1. A positive correlation was found instead between the ΔCD4 at T0 and the ΔCD4 at T1 (r = 0.603, p = 0.004).

**Fig 3:**
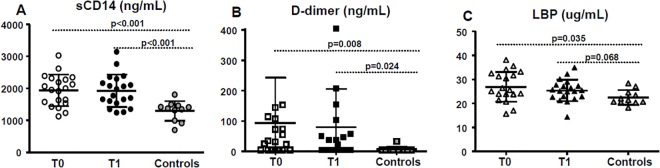
Plasma levels of sCD14, d-dimer and LBP. Scatter plots of sCD14 (A), d-dimer (B) and Lipopolysaccharide Binding Protein (LBP)(C) in patients before (T0) and after probiotics’ intake (T1) compared with controls. Horizontal bars in the scatter plot represent mean value with SD. P values <0.05 were considered statistically significant.

sCD14, D-dimer and LBP in HIV-infected patients treated with cART at T0 were significantly higher than the levels found in healthy donors (91% of the controls had undetectable level of D-dimer). These differences persist at T1 with the exception of LBP that normalizes itself, showing values similar to controls (p = 0.068) (Fig 3).

**Fig 4:**
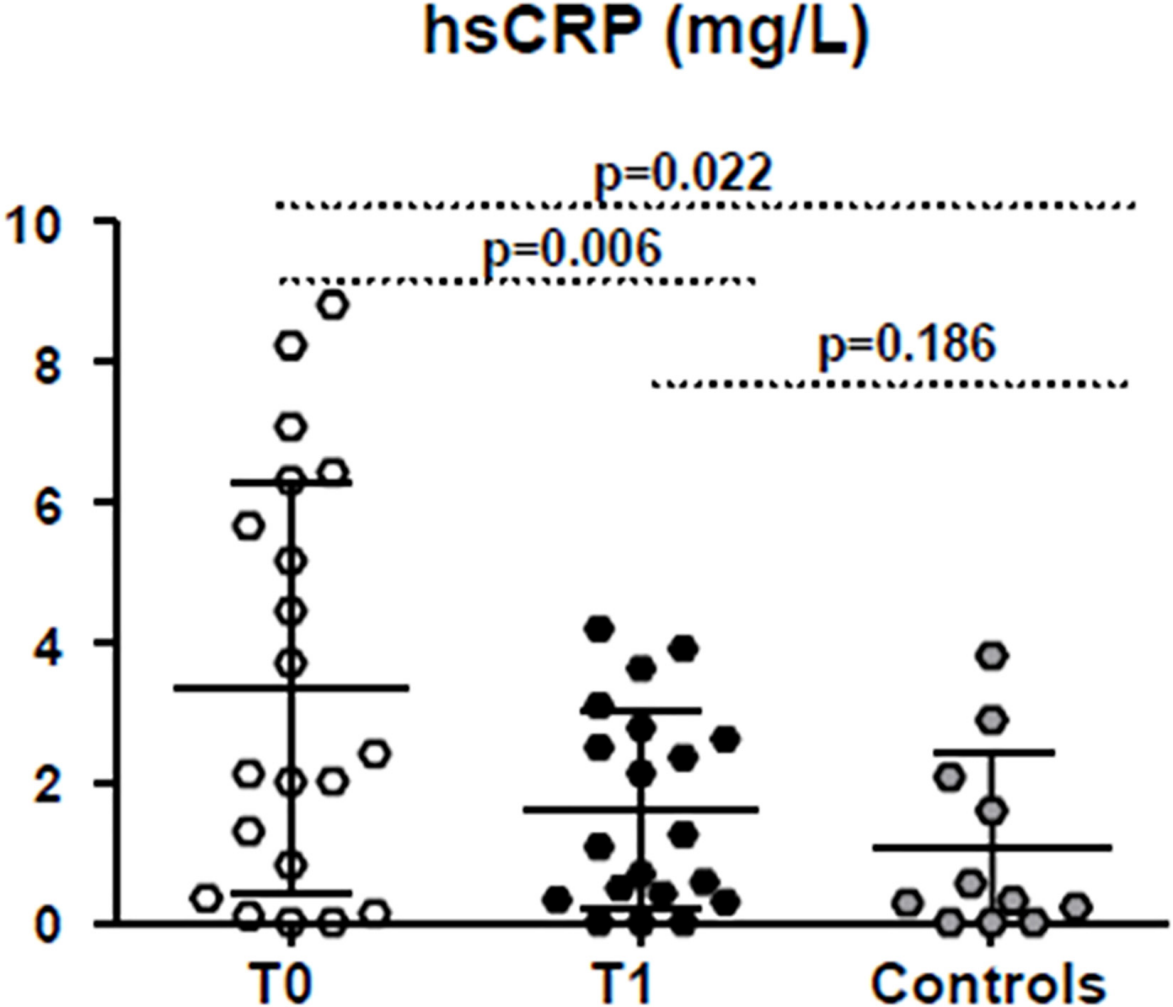
HsCRP plasma levels. High-sensitivity C-reactive protein (hsCRP) distribution in patients before (T0), and after probiotics’ assumption (T1) compared with controls Horizontal bars in the scatter plot represent mean value with SD. P values <0.05 were considered statistically significant.

IL-6 and CRP in HIV-1^+^ patients are similar to that found in control group at both observation times. On the contrary, the mean of hsCRP at T0 was 3.36±2.95mg/L and 1.625±1.42 mg/L at T1 (p = 0.006), becoming comparable to healthy donors (1.08±1.33, p = 0.186)(Fig 4).

By the Spearman’s rank sum test, the hsCRP level at T0 correlates positively with sCD14 (Correlation coefficient (CC):0.631, p = 0.002), IL-6 (CC:0.661, p = 0.001) and LBP (CC: 0.487, p = 0.029) and negatively with the D-dimer (CC:-0.553, p = 0.011). sCD14 and IL-6 are positively associated with Correlation coefficient 0.621 and p = 0.003.

These correlations disappear at the second time of analysis. On the other hand we observed a weakly significant correlation at T0 between sCD14 and D-dimer (p = 0.069) that persisted to T1 (CC: -0.484, p = 0.030).

Finally the patients declared in the questionnaire that they did not regularly consume the probiotics prior to enrollment in the study.

### Aging and immune activation

**Fig 5:**
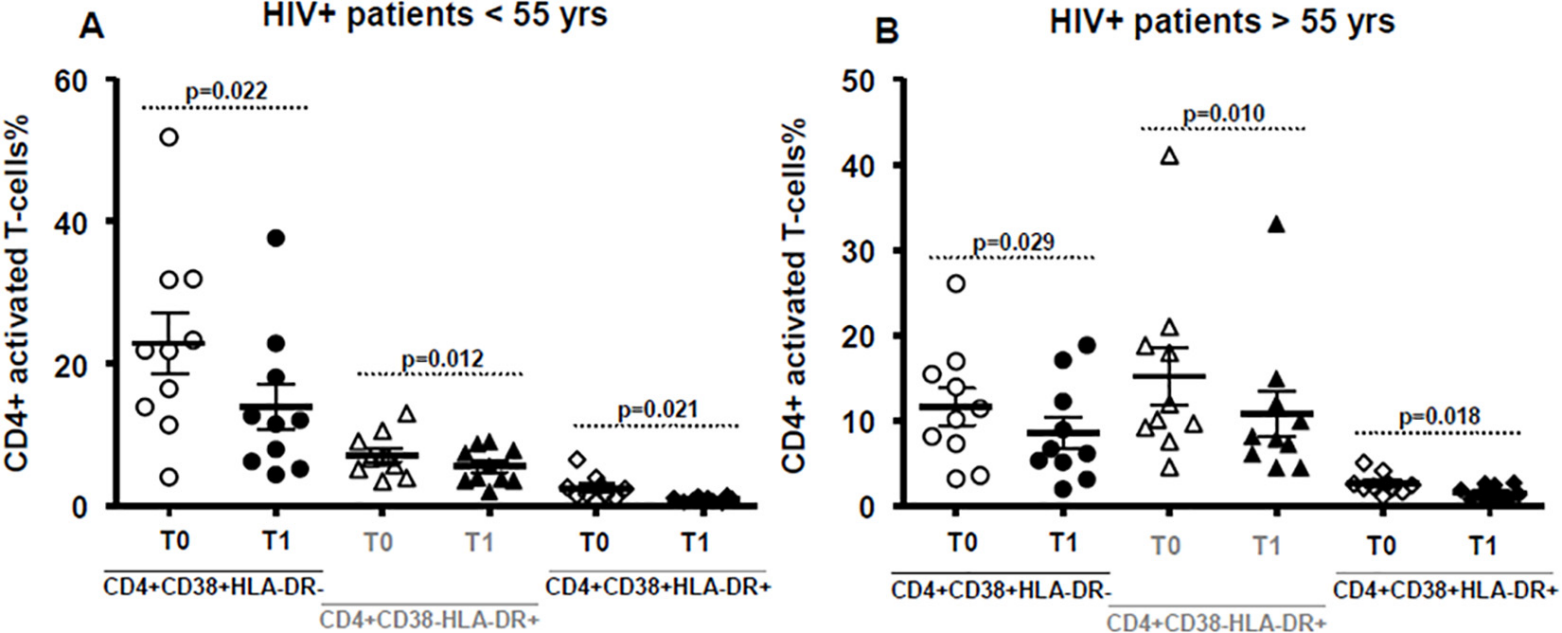
Levels of immune activation according to the age on CD4+ T-cells. Extent of expression and co-expression of activation markers CD38 and HLA-DR on CD4+ T-lymphocytes before (T0) and after probiotics’ intake (T1) in patients with less (A) and more (B) than 55 years. Horizontal bars in the scatter plot represent mean value with SD. P values <0.05 were considered statistically significant.

The sample was divided according to the median age, less or greater than 55 years old; the two groups were compared for levels of immune activation shown. There is differential activation of CD4 cells at T0: patients younger than 55 years show preferential activation of CD4+ CD38+ % T-cells subpopulation (22.85±13.37 vs 11.62±6.87 in the older group, p = 0.030) whereas there is a more pronounced activation of CD4+HLA-DR+ T-cells subpopulation in patients who are 55 years or older (15.19±10.53 versus 7.16±2.97 in the younger group, p = 0.032). Nevertheless, after taking probiotics, both groups show a statistically significant trend of reduction in the expression and co-expression of activation markers CD38 and HLA-DR on CD4 + T-lymphocytes, regardless of age (Fig 5).

However, the levels of CD4+CD38+HLA-DR+% T-cells shown by the older group at T1, are significantly higher than the other group (respectively 1.56±0.79 and 0.91±0.34, p = 0.027), thus showing a lower recovery. In this group CD4+CD38+HLA-DR+% T-cells fraction at T0 is also directly correlated to sCD14 levels (CC: 0.661, p = 0.033) and inversely related to d-dimer (CC: -0.644, p = 0.038). Moreover we observed a reduction of the levels of activated CD8 in both group, under and above 55 years, without statistically significant differences (Table 4).

**Table 4:** Immune activation markers’ mean values, standard deviations and paired T-test p-values according to the age of patients.

	MEAN T0 pts> 55 yrs	STD. DEV. T0 pts > 55 yrs	MEAN T1 pts> 55 yrs	STD.DEV.T1 pts > 55 yrs	PAIRED T-TEST p-valuespts > 55yrs	MEAN T0 pts< 55 yrs	STD. DEV. T0 pts < 55 yrs	MEAN T1 pts< 55 yrs	STD.DEV.T1 pts < 55 yrs	PAIRED T-TEST p-values pts < 55yrs
**CD4+CD38+HLA-DR-%**	11,63	6,87	8,56	5,73	**0,029**	22,85	13, 3	13,89	10,17	**0,022**
**CD4+CD38-HLA-DR+%**	15,19	10,53	10,83	8,46	**0,01**	7,17	2,98	5,57	2,50	**0,012**
**CD4+CD38+HLA-DR+%**	2,62	1,15	1,56	0,79	**0,018**	2,51	1,7	0,91	0,33	**0,021**
**CD8+CD38+HLA-DR-%**	5,60	4,04	3,07	5,07	0,264	4,35	3,25	3,51	4,21	0,657
**CD8+CD38-HLA-DR+%**	17,3	13,9	17,83	11,27	0,857	11,04	6,92	8,62	3,58	0,301
**CD8+CD38+HLA-DR+%**	6,18	5,47	3,69	2,82	0,187	4,28	3,13	1,87	1,08	0,074

In the group with less than 55 years, Interleukin 6 post-probiotic treatment falls to lower levels (5.97±6.48 pg/mL before and 1.81±2.00 pg/mL after, p = 0.037) and its levels are always positively correlated to sCD14 plasma values (CC: 0.732, p = 0.013 at T0; CC: 0.696, p = 0.022 at T1). In the older group is instead hsCRP to achieve a significant reduction, reaching a level of 1.26±1.37 mg/L at T1 (2.73±2.69 mg/L at T0, p = 0.014) with a persistent positive correlation with sCD14 (CC: 0.624, p = 0.048 at T0; CC: 0.644, p = 0.038 at T1).

#### Nadir and immune activation

The enrolled subjects were divided according to the CD4-nadir (less than or greater than 250 cells/μl).

There are no appreciable differences in the levels of immune activation between the two groups at T0 and T1, for both CD4 and CD8 T-cells.

The subjects with CD4 T-cells count greater than 250 (7 pts) have a significant decrease only of the activation markers CD38, on both CD4 (12.11±6.18 at T0 and 7.62±3.31 at T1, p = 0.028) and CD8 (3.86±1.82 at T0 and 1.72±1.52 at T1, p = 0.032).

**Fig 6:**
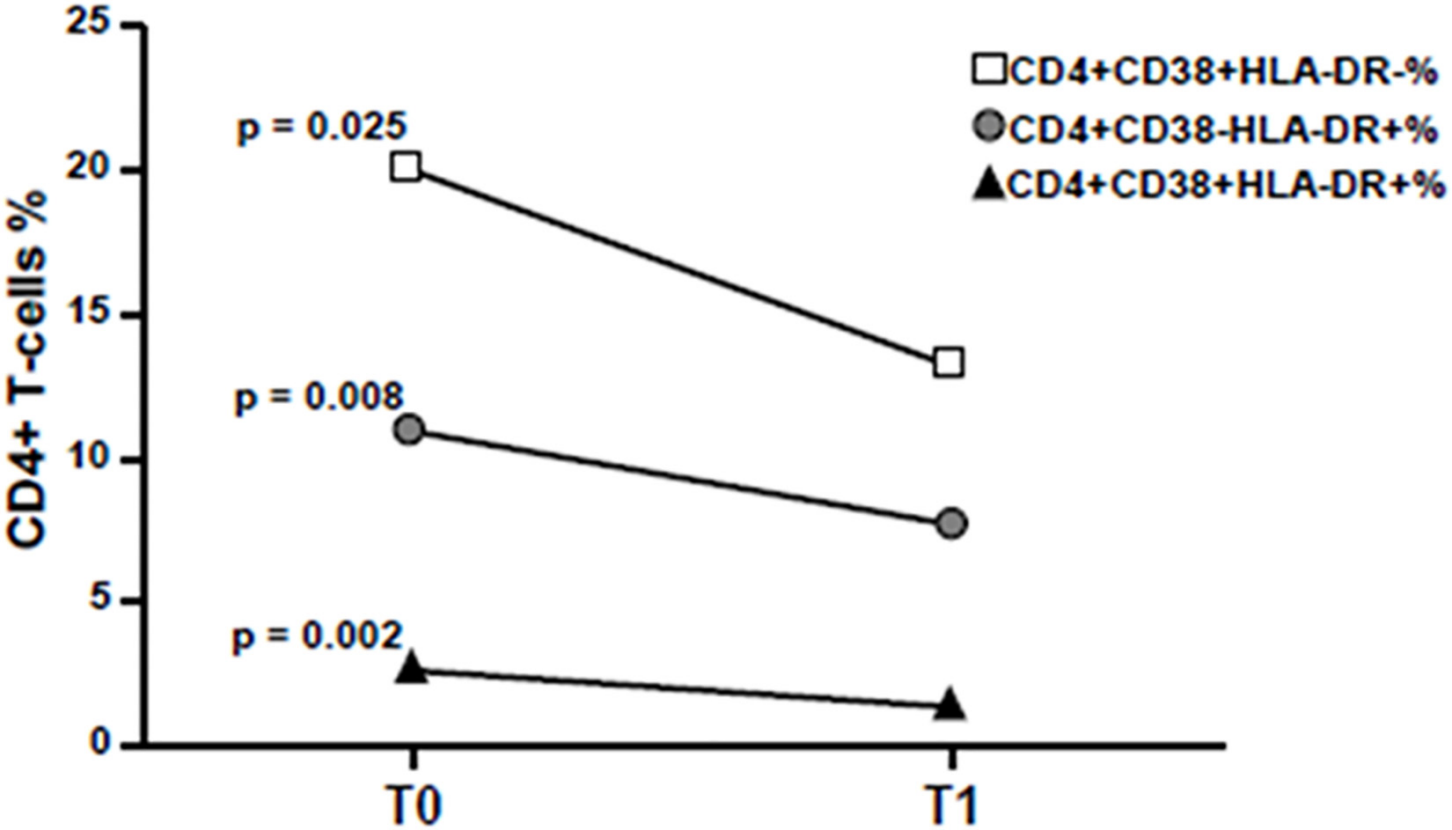
Effects of probiotics on T-cells activation in patients with CD4 count less than 250 cells/μl. Longitudinal assessment of CD38 and HLA-DR markers and their simultaneous expression on CD4 T cells in peripheral blood. in patients with a CD4 count less than 250 cells/μl at HIV infection diagnosis. P values <0.05 were considered statistically significant.

Those who had a CD4 count less than 250 cells/μL (13 patients) instead show a clear reduction in the levels of immune activation on CD4 T-lymphocytes, for both markers CD38 (20.00±13.39 at T0 and 13.17±9.85 at T1, p = 0.025) and HLA-DR (10.94±5.33 at T0 and 7.72±3.35 at T1, p = 0.008). The same trend was observed in the double activated CD4 subpopulation (2.66±1.10 at T0 and 1.39±0.74 at T1, p = 0.002) (Fig 6). No appreciable reduction was found for the activation of CD8+ T cells.

**Fig 7:**
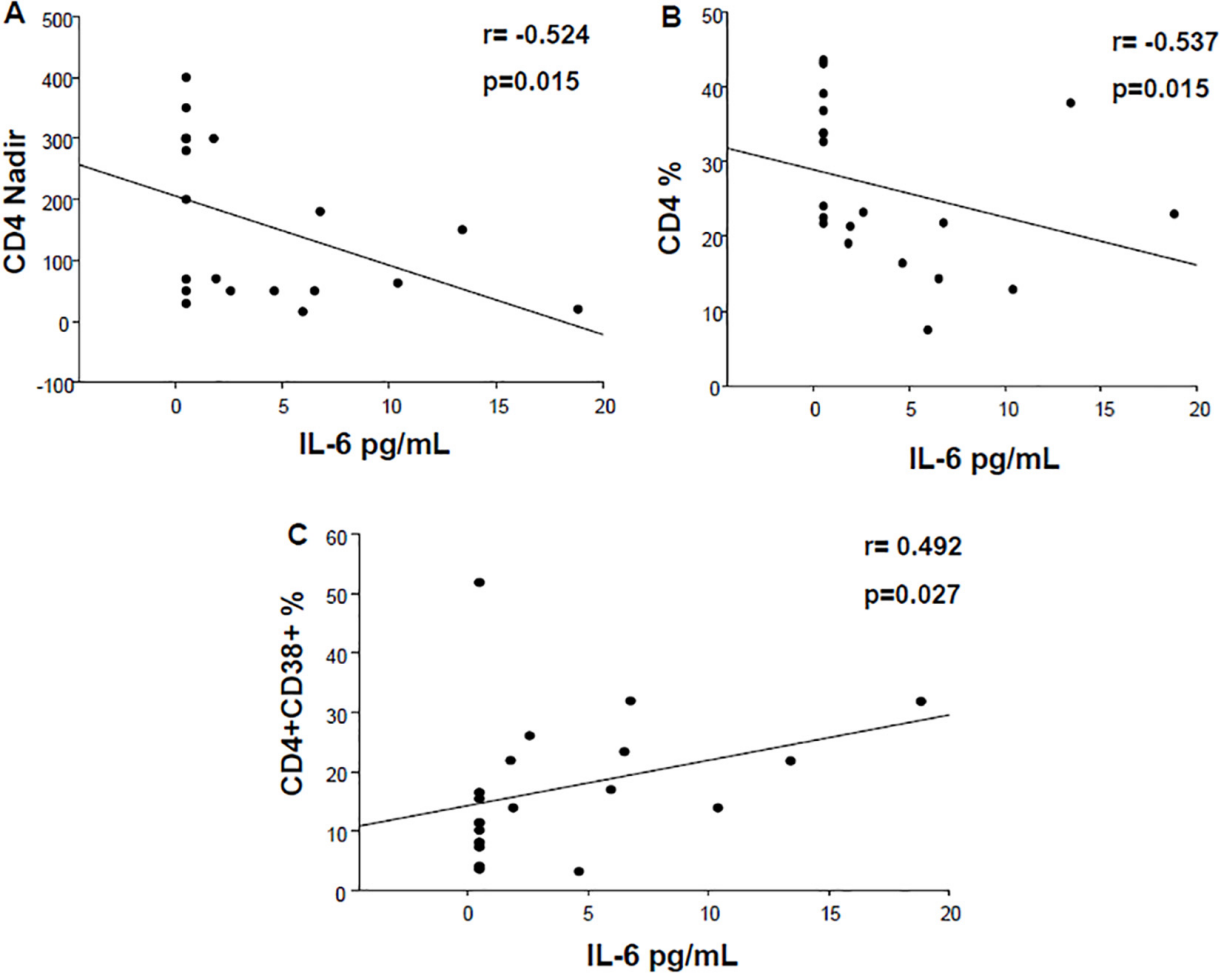
IL-6, nadir and immune activation at baseline. IL-6 levels at T0 were inversely correlated to CD4-Nadir (A) and CD4% (B), and positively correlated with the frequencies of activated, CD4+CD38+ T-cells (C). P values <0.05 were considered statistically significant; r represents correlation coefficient.

IL-6 levels at T0 were inversely correlated to CD4-Nadir (CC: -0.524, p = 0.015) and CD4% (CC: -0.537, p = 0.015), and positively correlated with the frequencies of activated, CD4+CD38+ T-cells (CC: 0.492, p = 0.027) (Fig 7). Although we observed that IL-6 levels were decreased after probiotic administration (p = 0.027), the negative correlation between CD4 and the levels of IL-6 only hold if pre-treatment (T0) data are included, presumably because this “pathology” is improved with the probiotics.

#### Years of infection and immune activation

Dividing the sample according to the years elapsed from infection (less than or greater than 10 years), we investigated the effective reduction of immune activation that occurs in the two groups and the difference between them.

Both groups start at comparable levels of CD4 T-cells immune activation (respectively 2.68±1.68 in the group infected by more time and 2.40±0.99 in the other group) and show similar value at T1 (respectively 1.37±0.8 and 1.03±0.42). However, the reduction in the levels of CD4+/CD38+/HLA-DR+ in the group infected for more years (12 patients) at T1 is 48.79% less than at T0, compared with a more marked reduction in the group recently infected (-56.86%).

Instead, the immune activation of CD8 T-cells (CD8+/CD38+/HLA-DR+) decreases only in those subjects infected by less than 10 years (8 patients), showing a reduction of 61.68% at T1 vs 36.60% in the other group.

We observed a positive correlation between the levels of D-dimer and years of infection (CC: 0.583, p = 0.014). This finding persist also after the assumption of probiotics (CC: 0.592, p = 0.006).

However, the sample is too small to appreciate significant correlations between the years of infection and the degree of immune-activation.

#### Physical benefits perceived by patients

After probiotics assumption all the patients enrolled reported beneficial effects consisting in substantial improvement in digestion, reduced intestinal bloating, reduction of acute diarrhoeal episodes or constipation; a small fraction of the subjects also stated reduction of allergic episodes and less fatigue. Only 29% of patients certifies that they have already taken in the past lactic acid bacteria and only a third of the sample regularly assumes yogurt.

The cost derived from the constant assumption of probiotics has been declared too expensive by all participants.

## Discussion

Chronic inflammation plays a central role in AIDS pathogenesis and contributes to morbidity and mortality in HIV-infected adults [15]. In the current study, we observed that in cART treated patients despite effective antiretroviral therapy the levels of the marker of immune activation were higher than healthy donors. All patients after 24 weeks probiotic supplementation showed a weak increase of CD4 T cells in the blood and a significant reduction of the levels of CD4+CD38+,CD4+HLA-DR+ and CD8+CD38+HLA-DR+ T cells. In addition also the expression of activation markers on CD4 and CD8 surface at T1 was comparable to healthy donors. The value of LBP was normalized after the assumption of probiotics and on the other hand the levels of IL-6, CRP was similar to that observed in both group at both time of observation. These results underline and highlight that the use of probiotic can be useful to the recovery of immunological responses in cART-treated individuals. To date is known the important role of the inflammation to induce non-AIDS related events and to cause the aging of the patients. The opportunity to integrate to cART probiotics intake to control the inflammation status could contribute to take the life expectancy comparable to health donors[20–22].

However, the suggestions provided by this pilot study and other researches on the topic are still preliminary and fragmentary to draw any firm conclusions. Instead they represent an important incentive to justify larger studies to assess whether probiotic administration truly could decrease inflammation and improve the prognosis of cART-treated, HIV-infected, individuals.

In our study we observed a significant reduction of hsCRP after probiotics intake and this marker is related to significant reduction of the cardiovascular diseases risk. In particular we observed that the percentage of patients with elevated hsCRP decreased significantly from 45% to 20% after the assumption of probiotics.

We did not find any significant correlation between both the nadir of CD4 T cells and the time of exposure to the virus and the levels of immune activation. However we observed that patients with CD4 T cells less than 250 cell/mL show a reduction of the levels of immune activation for CD38, HLA-DR and CD4 subpopulation. These data support that the value of CD4 less than 250 cell/μL is important to consider the risk of the progression of the infection. In fact by guidelines the value of 250 cell/μl is important to begin the primary prophylaxis for infectious disease such as pneumonia by *Pneumocystis jirovecii*.

The persistence of immune activation also after the recovery of the number of peripheral CD4 T cells, together to the continuous undetectable levels of HIV-RNA and to the long exposition to antiretroviral treatment suggests that could be useful combine the antiretroviral therapy to the cure of GALT dysbiosis. Anyway at present, few studies report the use of prebiotics or probiotics in HIV therapy. In available data, prebiotics were found to be effective in modulating gut microbiota reconstruction and their use resulted in decreased LPS and sCD14 levels and activation of CD4+ T-cell and NK cells. Prebiotics are used also to modulate resident gut homeostasis [23, 24] and selectively promote growth of beneficial bacterial species. In this way, pathogenic gut bacteria that maybe implicated in opportunistic infections, a hallmark of HIV infection, are eliminated or reduced. Also probiotics have safely been administered to HIV-infected individuals but their use resulted in modest improvements in CD4^+^ T cell counts and clinical GI symptoms [25, 26]. Adequately administered probiotics can express antimicrobial compounds that may kill or inhibit the growth of pathogenic microbial species in the gut, thus, conferring a net health benefit [27, 28]. Moreover probiotics regulate the intestinal epithelial barrier, in fact increases in tight junction proteins as a response to the administration of probiotics have been demonstrated in vivo and in vitro [29]. The contribution of probiotics in HIV disease management has been explored but conclusive knowledge is lacking.

The major limitation of this study is that this was not a randomized, controlled trial with a comparable HIV-infected control group: in fact the lack of a proper comparison group, specifically HIV-positive subjects not taking probiotics, should be considered an important limitation in the analysis of data.

Nevertheless we did measure parameters, longitudinally, in our treatment groups which would, presumably and at least partially, control for many environmental factors. In fact cART reduces T cell activation, but this parameter may remain abnormally elevated even after years of HIV-RNA undetectability. Persistent immune activation despite sustained antiretroviral therapy is associated with a blunted recovery of CD4+ T cells and augmented susceptibility to the development of HIV infection-associated comorbidities [30–33]. Also, previous works studying temporal reductions in immune activation after administration of cART showed how these levels tend to plateau after several months of treatment. Therefore, we feel confident that our data measured a probiotic effect. Also, as mentioned above, the control group did not consume probiotics.

Considering the large number of potential confounders inherent in the study design, a proper multivariate analysis should be more appropriate but was not possible to do for the small sample size. Another potential caveat of this study is that the assessment of immune activation in peripheral blood was conducted only by determining the number of CD4+CD38+,CD4+HLA-DR+ and CD8+CD38+HLA-DR+ T cells. Vice versa we do not think that the previous occasional probiotic consumption (as declared by patients in the questionnaire) influenced the results obtained in the present study. Finally, we underline that the use of questionnaire to assess the actual intake of probiotics is a simple way to know the adherence of patients and could represent an important bias, but unfortunately we were not able to measure directly the level of probiotics in the stools of patients.

In conclusion current and emerging studies support the concept that probiotic bacteria can provide specific benefit in HIV infected patients during antiretroviral treatment by improvement of the microbiota and reducing mucosal and systemic inflammation [25, 34].

However further longitudinal studies of the effects of the probiotics on the GALT will be necessary to clarify the relationship between the persistence of immune activation in patients with fully suppressed plasma viraemia.

## Supporting Information

S1 Data Database of the study. (XLS)

S1 Protocol Study Protocol in Italian language (original protocol). (DOC)

S2 Protocol Study Protocol in English language (translated protocol). (DOC)

S1 TREND Checklist TREND Statement Checklist. (DOC)
