# Supplementary material for: Probiotics Reduce Inflammation in Antiretroviral Treated, HIV-Infected Individuals: Results of the “Probio-HIV” Clinical Trial
Source: PLoS One. 2015 Sep 16;10(9):e0137200. doi: 10.1371/journal.pone.0137200 (PMC4573418; doi:10.1371/journal.pone.0137200)
Supplement: S1 Protocol — (DOC) [file pone.0137200.s002.doc]

Roma 12/12/2012

**Dipartimento di Sanità Pubblica e Malattie Infettive**

*Effetti della traslocazione microbica e dei probiotici sui marcatori di attivazione immunitaria nei pazienti HIV positivi in HAART e studio degli effetti extra-virologici della terapia.*

**INTRODUZIONE**

Durante la fase acuta dell'infezione da HIV, vi è una significativa perturbazione ai componenti immunologici e strutturali del tratto gastrointestinale (GI). La massiccia deplezione delle cellule T CD4 del tratto GI, basse frequenze di CD4 e CD8 T producenti IL-17 (Macal *et al.,* 2008), apoptosi degli enterociti (con conseguente danno alla barriera strutturale del tratto GI) e l'aumento della permeabilità intestinale, sono tutte manifestazioni della progressiva infezione da HIV negli esseri umani. Inoltre, la sistemica e generalizzata attivazione del sistema immunitario è una caratteristica della fase cronica della progressiva infezione HIV / SIV, e il grado in cui il sistema immunitario viene attivato è il miglior predittore del tasso di progressione della malattia. Infatti, una delle cause di attivazione immunitaria è l’aumentata traslocazione microbica (MT) a causa del danneggiamento del tratto gastrointestinale: livelli elevati di LPS sono stati trovati nel plasma di individui cronicamente affetti da HIV rispetto a soggetti con infezione acuta o non infetti. Coerentemente con questo ruolo pro infiammatorio di LPS nella circolazione sistemica, i livelli di LPS nel plasma sono stati descritti come indicatori di attivazione immunitaria di entrambe le componenti del sistema immunitario, innata e adattiva (Douek e Brenchley, 2012).

**I OBIETTIVO**

L’obiettivo dello studio è quello di stimare e comprendere gli effetti della traslocazione microbica sulla circolazione periferica andando a valutare:

1. l’immunoattivazione
2. I livelli plasmatici dei markers di traslocazione microbica (LPS, sCD14, LBP, EndoCAb) e di infiammazione (hs-CRP, IL-6, TNFa)
3. la secrezione di TNF-α e IL-1β da parte dei monociti, basale e in seguito a stimolo con LPS
4. la secrezione di IL-17 dei linfociti T CD4+

**METODI**

Saranno arruolati pazienti naive e in terapia HAART con HIV-RNA < 50 cp/mL.

Il protocollo necessita di 1 provetta viola (EDTA) e 1 verde (eparina) sulle quali si procederà con le seguenti analisi:

1. raccolta del plasma per successive analisi immunoenzimatiche (test ELISA) per rilevare i livelli plasmatici dei markers di traslocazione microbica e di infiammazione.
2. Marcatura di superficie per valutare il grado di immunoattivazione su sangue intero (CD3, CD4, CD8, CD38 e HLA-DR)
3. Incubazione 4 ore a 37°C con/senza LPS (100ng/mL) e successiva marcatura intracellulare su sangue intero per valutare in termini percentuali i monociti (CD45+CD14+HLA-DR+) secernenti TNF-α e IL-1β.
4. Incubazione 5 ore a 37°C con/senza stimolazione aspecifica (PMA e Ionomicina) e successiva marcatura intracellulare su sangue intero per valutare in termini percentuali i linfociti T CD4+ secernenti IL-17.

Tutte le analisi saranno effettuate al tempo zero e al tempo 1, dopo circa 6 mesi di assunzione volontaria di probiotici (Yovis, fermenti lattici).

Sugli stessi pazienti, il nostro secondo obiettivo è andare a studiare gli effetti extra-virologici della terapia HAART, in particolar modo la nostra attenzione sarà rivolta alla classe degli inibitori delle proteasi (PI). Infatti l’impiego di questi ultimi ha portato ad un netto miglioramento dello stato immunologico dei pazienti permettendo il recupero dei linfociti T CD4+ a livelli non raggiungibili con i farmaci utilizzati in precedenza.

Sui primi PI introdotti nella pratica clinica sono stati già descritti effetti anti-apoptotici (*Badley AD et all,1998*) ed effetti inibitori diretti sulle aspartil proteasi della *Candida albicans* e dello *Pneumocystis carinii* che rappresentano degli importanti fattori di virulenza per questi funghi (*Pati S et all, 2002 Phenix BN et all, 2001*). Gli effetti diretti dei farmaci potrebbero svolgere un ruolo importante nell’ottica della immunomodulazione che in corso di infezione da HIV potrebbe concorrere a ridurre lo stato di immunoattivazione cronica.

**II OBIETTIVO**

Non ci sono studi che riguardano i PI di recente introduzione; sulla base di quanto esposto l’obiettivo principale del progetto di ricerca è estendere i nostri studi sul darunavir (DRV), un PI di seconda generazione, indicato per il trattamento di pazienti che hanno fallito più di un trattamento terapeutico a base di altri inibitori delle proteasi.

**METODI**

**EFFETTI SULL’APOPTOSI CELLULARE**

Per ogni paziente il protocollo necessita di due provette di sangue eparinato, sulle quali verranno eseguite le seguenti analisi:

1. Separazione dei linfo-monociti utilizzando il protocollo di separazione su gradiente di densità Ficoll-Paque^TM^ PLUS.
2. Dopo la separazione su Ficoll-Paque^TM^ PLUS, i PMN verranno recuperati con ulteriore separazione mediante destrano 6%.
3. Le cellule (1x10^6^/ml) verranno colorate con gli anticorpi monoclonali CD4-APC, CD8-PE e con Annexina V/Ioduro di propidio e dopo una incubazione di 20 minuti e un lavaggio con una soluzione di Binding le cellule verranno analizzate al citofluorimentro.

**EFFETTI SULLA FUNZIONALITA’ CELLULARE**

Verranno effettuati sugli stessi pazienti studi ex-vivo sulla capacità chemiotattica di linfociti T, monociti/macrofagi e PMN e verrà inoltre testata la capacità di produrre citochine e chemochine coinvolte nell’attivazione immunitaria.

1. Su tutti i comparti cellulari verrà stimata la chemiotassi mediante camere di Boyden utilizzando diversi chemioattrattanti specifici in funzione del comparto cellulare.
2. Verrà studiata l’attività funzionale di linfociti, monociti e PMN.
   - Linfociti: la produzione di citochine pro e antiinfiammatorie quali IL2, IL 6, IFN-γ, IL10, IL12 verrà valutata su cellule stimolate con PHA, LPS.
   - Monociti e PMN: l’attività di killing di queste cellule verrà stimata mediante il test di candidocidia.

**STATISTICA**

Determinazione della dimensione del campione e metodi statistici:

La determinazione della dimensione del campione non è possibile in questo studio pilota in quanto non sono disponibili in letteratura dati di popolazione riguardo all’obiettivo primario dello studio. Verranno pertanto studiati in via preliminare 20 pazienti HIV positivi, in una fase precedente l'inizio della assunzione del probiotico e a 6 mesi. Come gruppo di controllo saranno utilizzati 10 soggetti HIV negativi. Sui dati raccolti, relativi alle eventuali modificazioni presentate dai singoli pazienti dei due gruppi, verrà effettuata l’analisi descrittiva.

**BIBLIOGRAFIA**

- Brenchley JM, Douek DC. 2012. *Annu. Rev. Immunol.* 30:149-173
- Macal M, Sankaran S, Chun TW, Reay E, Flamm J, Prindiville TJ, Dnadekar S. 2008. Effective CD4 + T-cell restoration in gutassociated lymphoid tissue of HIV-infected patients is associated with enhanced Th17 cells and polyfunctional HIV-specific T-cell responses. *Mucosal Immunology* vol. 1, 6: 475

Badley AD, Dockrell DH, Algeciras A et al (1998) *in vivo* analysis of Fas/FasL interactions in HIV-infected patients J Clin Invest 102(1):79–87.

[Pati S, Pelser CB, Dufraine J, Bryant JL, Reitz MS Jr, Weichold FF.](http://www.ncbi.nlm.nih.gov/pubmed/11986235?ordinalpos=5&itool=EntrezSystem2.PEntrez.Pubmed.Pubmed_ResultsPanel.Pubmed_RVDocSum) Antitumorigenic effects of HIV protease inhibitor ritonavir: inhibition of Kaposi sarcoma. Blood. 2002;99:3771-9.

[Phenix BN, Lum JJ, Nie Z, Sanchez-Dardon J, Badley AD.](http://www.ncbi.nlm.nih.gov/pubmed/11493454?ordinalpos=2&itool=EntrezSystem2.PEntrez.Pubmed.Pubmed_ResultsPanel.Pubmed_RVDocSum) Antiapoptotic mechanism of HIV protease inhibitors: preventing mitochondrial transmembrane potential loss. Blood. 2001; 98:1078-85.
