# Supplementary material for: Probiotics Reduce Inflammation in Antiretroviral Treated, HIV-Infected Individuals: Results of the “Probio-HIV” Clinical Trial
Source: PLoS One. 2015 Sep 16;10(9):e0137200. doi: 10.1371/journal.pone.0137200 (PMC4573418; doi:10.1371/journal.pone.0137200)
Supplement: S2 Protocol — (DOC) [file pone.0137200.s003.doc]

*UNIVERSITY OF ROME SAPIENZA*

*Department of Public Health and Infectious Diseases*

**Rome 12/12/2012**

**Effects of microbial translocation and probiotics on markers of immune activation in HIV-positive patients on HAART and non-virological study of the effects of antiretroviral therapy.**

**INTRODUCTION**

During the acute phase of HIV infection, there is a significant disturbance of the immunological and structural components of the gastrointestinal tract (GI). The massive depletion of CD4 T cells of the GI tract, low frequencies of CD4 and CD8 T-producing IL-17 (Macal et al., 2008), apoptosis of enterocytes (resulting in structural damage to the barrier of the GI tract) and increased intestinal permeability, are all manifestations of progressive HIV infection in humans. Furthermore, the systemic and generalized activation of the immune system is a characteristic of the chronic phase of progressive HIV infection / SIV; moreover the degree of activation of the immune system is the best predictor of the progression rate of the disease. In fact, one of the causes of immune activation is the increased microbial translocation (MT) because of damage of the gastrointestinal tract: high levels of LPS were found in the plasma of individuals chronically infected with HIV compared to subjects with acute infection or uninfected. Consistent with this pro-inflammatory role of LPS in the systemic circulation, the levels of LPS in plasma have been described as indicators of immune activation of both components of the immune system, innate and adaptive immunity (Douek and Brenchley, 2012).

**I PRIMARY OBJECTIVES**

The goal of the study is to assess and understand the effects of microbial translocation on peripheral blood. For this reason we will evaluate:

1) the immune activation

2) the plasma levels of markers of microbial translocation (LPS, sCD14, LBP, EndoCAb) and inflammation (hs-CRP, IL-6, TNFa)

3) secretion of TNF-α and IL-1β by monocytes, at baseline and after stimulation with LPS

4) the secretion of IL-17 lymphocyte T CD4 +

***METHODS***

HIV positive, HAART-naive patients and HIV positive on effective HAART therapy subjects (HIV-RNA <50 copies / mL) will be enrolled.

The protocol requires 1 EDTA supplemented tube and 1 heparin supplemented tube on which we will proceed with the following analysis:

1. plasma collection and subsequent immunoassays analysis (ELISA) to detect plasma levels of markers of microbial translocation and inflammation.

2. to mark cell surface for assessing the degree of immune activation in whole blood (CD3, CD4, CD8, CD38 and HLA-DR)

3. Incubation for 4 hours at 37 ° C with / without LPS (100 ng / mL) and subsequent intracellular marking to assess the percentage amount of monocytes (CD45 + CD14 + HLA-DR +) secreting TNF-α and IL-1β.

4. Incubation 5 hours at 37 ° C with / without non-specific stimulation (PMA and Ionomycin) and subsequent intracellular marking of whole blood to assess the percentage amount of the CD4 + T cells secreting IL-17.

All analyzes will be performed at baseline and after about 6 months of the start of the intake of probiotics in HIV positive patients.

On the same patients, our second goal is to study the effects of the extra-virological HAART, in particular, our attention will be focalized on the class of protease inhibitors (PI).

The direct effects of these drugs could play an relevant role in the immunomodulation and help to reduce the state of chronic immune activation.

**II SECONDARY OBJECTIVES**

There are no studies concerning the recent introduction of PI; on the basis of the above, the main objective of the research project is to extend our studies on darunavir (DRV), a second-generation PI, indicated for the treatment of patients who have failed more than a therapeutic treatment based on other inhibitors proteases.

***METHODS***

EFFECTS ON CELLULAR APOPTOSIS

For each patient, the protocol requires two tubes of heparinized blood, on which we will perform the following analyzes:

1. Separation of lympho-monocytes using the protocol of separation density gradient Ficoll-PaqueTM PLUS.

2. After separation on Ficoll-PaqueTM PLUS, the PMN will be retrieved with further separation by dextran 6%.

3. The cells (1x10^6^ / ml) will be stained with monoclonal antibodies CD4-APC, CD8-PE and with Annexin V / Propidium Iodide. Cells will be analyzed by cytofluorimetric analysis after an incubation of 20 minutes and a wash with a solution of Binding.

**EFFECTS ON FUNCTIONALITY OF CELL**

Ex vivo studies i) on the chemotactic ability of T lymphocytes, monocytes / macrophages and PMN and ii) on the ability to produce cytokines and chemokines involved in activating immune will be performed.

1. On all cellular compartments we will estimate chemotaxis through Boyden chambers using different chemoattractants.

2. We will investigate the functional activity of lymphocytes, monocytes and PMN:

- lymphocytes: the production of pro and anti-inflammatory such as IL2, IL 6, IFN-gamma, IL10, IL12 will be evaluated on cells stimulated with PHA, LPS.

- monocytes and PMN: the killing activity of these cells will be estimated by testing candidocidia.

**STATISTICS**

Determination of sample size and statistical methods:

The determination of the sample size is not possible in this pilot study because data of the population about the primary objective of the study are not available in the literature. Therefore we will study 20 HIV-positive patients before the start of the intake of probiotic and after 6 months. As control group 10 HIV-negative subjects will be used. On the data collected will be performed a descriptive analysis.

**REFERENCES**

- Brenchley JM, Douek DC. 2012. *Annu. Rev. Immunol.* 30:149-173
- Macal M, Sankaran S, Chun TW, Reay E, Flamm J, Prindiville TJ, Dnadekar S. 2008. Effective CD4 + T-cell restoration in gutassociated lymphoid tissue of HIV-infected patients is associated with enhanced Th17 cells and polyfunctional HIV-specific T-cell responses. *Mucosal Immunology* vol. 1, 6: 475

Badley AD, Dockrell DH, Algeciras A et al (1998) *in vivo* analysis of Fas/FasL interactions in HIV-infected patients J Clin Invest 102(1):79–87.

Pati S, Pelser CB, Dufraine J, Bryant JL, Reitz MS Jr, Weichold FF. Antitumorigenic effects of HIV protease inhibitor ritonavir: inhibition of Kaposi sarcoma. Blood. 2002;99:3771-9.

Phenix BN, Lum JJ, Nie Z, Sanchez-Dardon J, Badley AD. Antiapoptotic mechanism of HIV protease inhibitors: preventing mitochondrial transmembrane potential loss. Blood. 2001; 98:1078-85.
